# Molecularly Imprinted Polymers for Gossypol via Sol–Gel, Bulk, and Surface Layer Imprinting—A Comparative Study

**DOI:** 10.3390/polym11040602

**Published:** 2019-04-02

**Authors:** Lulu Wang, Keke Zhi, Yagang Zhang, Yanxia Liu, Letao Zhang, Akram Yasin, Qifeng Lin

**Affiliations:** 1 Xinjiang Technical Institute of Physics and Chemistry, Chinese Academy of Sciences, Urumqi 830011, China; wanglulu@ms.xjb.ac.cn (L.W.); zhikeke@ms.xjb.ac.cn (K.Z.); liuyanxia@ms.xjb.ac.cn (Y.L.); zhanglt@ms.xjb.ac.cn (L.Z.); akram@ms.xjb.ac.cn (A.Y.); 2 University of Chinese Academy of Sciences, Beijing 100049, China; 3 Department of Chemical and Environmental Engineering, Xinjiang Institute of Engineering, Urumqi 830026, China; 4 Xinjiang Institute of Ecology and Geography, Chinese Academy of Sciences, Urumqi 830011, China; lqf@ms.xjb.ac.cn

**Keywords:** gossypol, bulk polymerization, sol–gel surface imprinting, adsorption kinetics, adsorption isotherms, adsorption selectivity

## Abstract

Three gossypol molecularly imprinted polymers (MIPs) were prepared by bulk polymerization (MIP1), surface layer imprinting using silica gel as the support (MIP2), and the sol-gel process (MIP3). The as-prepared MIPs were characterized by SEM and nitrogen adsorption−desorption techniques to study the morphology structure. The adsorption experiments exhibited that MIP1 had adsorption capacity as high as 564 mg·g^−1^. The MIP2 showed faster adsorption kinetics than MIP1 and MIP3. The adsorption equilibrium could be reached for gossypol in 40 min. A selectivity study showed that the adsorption capacity of MIPs for gossypol was about 1.9 times higher than that of the structurally-similar analogs ellagic acid and 6.6 times higher than that of the quercetin. It was found that the pseudo-second-order kinetic model and the Freundlich isotherm model were more applicable for the adsorption kinetics and adsorption isotherm of gossypol binding onto the MIP1 and MIP2, respectively. Results suggested that among those three, the MIP2 was a desirable sorbent for rapid adsorption and MIP1 was suitable for selective recognition of gossypol.

## 1. Introduction

Gossypol is a toxic compound indigenous to the seeds of cotton plants. Free gossypol can cause antifertility effects, growth depression, and other side effects in mammals [1,2,3,4,5]. As a by-product of the cotton industry, cottonseeds have become important feed resources because of their high nutritional quality. China is the second largest cotton producer in the world. Cottonseed meal is an important source of high-quality yet inexpensive protein feed for animal husbandry and aquaculture. However, its application is limited due to the presence of toxic gossypol in cottonseed. Thus, discovering methods for detoxifying gossypol in cottonseed related products have become an urgent task.

For this purpose, various approaches for removing gossypol have been developed, including microbial fermentation [6], solvent extraction [7], ferrous sulfate treatment [8], and adsorption with materials [9,10,11,12]. Among these, adsorption is considered as a feasible and viable method because of its mild operation conditions and cost-effectiveness. Some adsorbents have been reported for the removal of gossypol, such as alumina, silica and synthetic magnesium silicates [11,12], and molecularly imprinted polymers (MIPs) [9]. In comparison to other adsorbents, MIPs feature high affinity and high efficiency for the recognition and removal of gossypol.

MIPs are versatile and tailor-made recognition materials that are designed to be able to recognize a specific template molecule [13,14,15,16,17]. MIPs form highly cross-linked polymeric networks by polymerizing functional monomers and cross-linkers along with a target molecule, usually called a template. In the pre-polymerization process, functional monomers with recognition groups interact with a template molecule through noncovalent or covalent interactions to form a pre-polymerization complex. Following polymerization with a high proportion of a cross-linker, the complex becomes fixed in its conformation. After removing the template molecule, the cavities are produced, featuring a specific three-dimensional shape as well as a chemical memory of the template so as to render the MIPs’ abilities of high affinity and selectivity for the template molecules. MIPs have drawn considerable attention for their high selectivity, cost-effectiveness, mechanical robustness, and chemical inertness. Although they are far from being as good as their biological counterparts such as enzymes and antibodies, MIPs are sometimes called synthetic enzymes and manmade antibodies [18]. MIPs have found applications in stationary phase extractions, sensors, separation, drug delivery, and biomimetic catalysts [19,20,21,22,23,24,25,26].

In the non-covalent approach, MIPs are usually prepared by bulk polymerization. MIPs made with bulk polymerization typically exhibit poor binding site accessibility. The highly cross-linked monolith type structure leads to incomplete removal of the template and even some trapped “dead” sites. It is also limited by slow mass transfer. In addition, the grinding after polymerization yields heterogeneous particles in terms of shapes and size. In order to obtain uniformly sized particles, sieving has to be used, and this process is known to cause loss of prepared materials. 

The surface molecular imprinting strategy is another approach proposed for developing MIPs. It is known to be able to generate a thin layer of imprinted materials on the surface of the support [27,28,29,30,31,32,33,34]. By doing it this way, the prepared recognition sites are positioned near the surface within the channels and cavities. This would be beneficial and desirable for the efficient removal of templates and low defusing resistance, and it improves the accessibility of those sites in the rebinding process. Additionally, surface MIPs tend to be more physically robust due to the presence of the support. The support plays a key role for the performance of MIPs. Silica gel particles are established, and there exists popular support for surface imprinting due to the chemically stable non-swelling properties, ease of modification, and satisfactory compatibility with aqueous and biological systems [35,36,37,38,39]. 

Few MIPs using gossypol as the template were reported. In the work reported by Zhao et al. [40], a two-layer structure of poly(methacrylic acid)/SiO_2_ bulks was designed and prepared for the detection of gossypol. The other two were developed by Zhang et al. [9,41] for the recognition and adsorption of gossypol. Recently, MIPs via the sol-gel process have been prepared [42,43,44,45,46,47,48,49]. However, a systematical study comparing bulk polymerization, sol-gel, and surface molecular imprinting has not been reported. It would be very helpful in practical applications to understand the strength and weakness of each above mentioned approach, especially for an interesting and important template such as gossypol.

For the above mentioned reasons, three MIPs were prepared using gossypol as the template via bulk polymerization, sol-gel, and surface molecular imprinting. Namely, MIP1 was prepared with dimethylaminoethyl methacrylate (DMAEMA) as the functional monomer (FM) and ethylene glycol dimethacrylate (EGDMA) as the cross-linker by bulk thermal polymerization [9]. MIP2 was prepared by combining the advantages of surface imprinting and the sol−gel process. Silica gel was chosen as the support for surface imprinting, and (3-aminopropyl)triethoxysilane (APTES) and Tetraethoxysilane (TEOS) were used as the functional monomer and the cross-linker. As a control, MIP3 without silica support was prepared under the same reaction condition as that of MIP2. The prepared MIPs and non-imprinted polymers (NIPs) were characterized by SEM and surface measurement. The binding performances of the MIP1, MIP2, and MIP3 for the recognition and adsorption of gossypol were evaluated by static adsorption experiments. The adsorption kinetics, adsorption isotherms, adsorption selectivity, adsorption capacity, as well as the reusability of the obtained MIPs were also investigated. 

## 2. Materials and Methods

### 2.1. Materials and Reagents

Gossypol was purchased from Sigma-Aldrich (Shanghai, China). Dimethylaminoethyl methacrylate (DMAEMA), ethylene glycol dimethacrylate (EGDMA), divinylbenzene (DVB), 1,1’-bi-2-naphthol, and 2,2-azobis(isobutyronitrile) (AIBN) were purchased from Adamas Reagent Co., Ltd. (Shanghai, China). Hexaphenol was purchased from WAKO Pure Chemical Industry, Ltd. (in Japan). Tetraethoxysilane (TEOS), ellagic acid, and quercetin were obtained from Adamas (Shanghai, China). Silica beads (0.5 μm average particle size) and (3-aminopropyl) triethoxysilane (APTES) were purchased from Alfa Aesar (Shanghai, China). Methanol, acetone, hydrochloric acid, acetic acid, and sodium hydroxide were all analytical grade and purchased from Tianjin Zhiyuan Chemical Co., Ltd. (Tianjin, China). Water was purified by a Millipore Milli-Q gradient system (Massachusetts, America) to HPLC grade.

### 2.2. Preparation of MIPs and NIPs

#### 2.2.1. Synthesis of MIP1 and NIP1 by Bulk Polymerization

Synthesis of the MIP1 [9]: Synthesis of the MIP1 by bulk polymerization followed a reported procedure. In general, gossypol (0.083 mmol, 43 mg), DMAEMA (1.0 mmol, 157 mg), EGDMA (5.0 mmol, 990 mg), and AIBN (0.27 mmol, 44 mg) were dissolved in 4.0 mL dichloromethane in a screw-capped vial. This mixture was sonicated for 5 min and degassed for 15 min under nitrogen. The vial was sealed and then immersed in a water bath at 65 °C for 24 h. As the control, a non-imprinted polymer (NIP1) was synthesized following the same protocol but without adding the template. The resulting polymer monoliths were crushed and ground with a mortar and pestle to a fine powder. The powder was sieved using 200 mesh sieve. Particle size fraction below 75 μm was collected. 

Removal of template molecule: For MIP1, the un-reacted species and templates were removed by Soxhlet extraction with methanol for 24 h and then washed with 10.0 mmol L^−1^ NaOH aqueous solutions for 2 h per cycle until no template molecules were detected by a UV-Vis spectroscopy. Finally, the MIPs were washed with pure water to be neutral. For NIPs, the particles were washed by Soxhlet extraction with methanol for 24 h to remove the un-reacted substances. Finally, both MIP and NIP were dried at 60 °C using the vacuum drying in an oven overnight. 

#### 2.2.2. Synthesis of MIP2 and NIP2 by Surface Layer Imprinting

Activation of silica gel: 8.0 g of silica was mixed with 60 mL of 33% (*w*/*w*) methanesulfonic acid and refluxed for 8 h at 110 °C with stirring. Following this, the activated silica gel was obtained by filtration, washed with pure water to neutralize, and then dried under vacuum at 70 °C for 10 h.

Synthesis of the MIP2: Gossypol (0.083 mmol, 300 mg) was dissolved in 3.0 mL of acetone under stirring, and 0.29 mL (8.5 mmol) of APTES was added into the mixture under stirring for 30 min to obtain the completely self-assembled complex with gossypol. Then, 58 mg of activated silica gel was added by stirring for 30 min to provide silica particles fully dispersed in solution. Then, 0.58 mL (2.6 mmol) of TEOS was added. The mixture was stirred for 5 min, and then 0.15 mL of 1.0 mol·L^−1^ acetic acid was added. The polymerization reaction was carried out at room temperature under stirring for 24 h to obtain particles with a high cross-linking structure. Finally, the particles were isolated by centrifugation at 8000 rpm for 5 min. As the control, a non-imprinted polymer (NIP2) was prepared and treated in an identical manner but in the absence of gossypol. 

Removal of template molecule: For MIP2, to remove the gossypol, the obtained polymers were repeatedly washed with 50 mL of a mixture of methanol and 6 mol·L^−1^ HCl (1:1, *v*/*v*) until no template molecules were detected by UV-Vis spectroscopy. Then, it was neutralized with 0.1 mol·L^−1^ NaOH solution and washed to neutral pH by pure water and methanol. For NIPs, the particles were washed to neutral pH with pure water and methanol to remove any un-reacted substances. Finally, both MIP and NIP were dried at 80 °C for 12 h under vacuum.

#### 2.2.3. Synthesis of MIP3 and NIP3 by Sol-Gel Process

In comparison, the MIP3 and NIP3 without the added silica gel support were prepared following the same protocols as for MIP2 and NIP2. Details for the preparation of MIPs and NIPs and the polymerization conditions are summarized in Table 1.

### 2.3. Characterization

The adsorption test was carried out using a UV-Vis spectrophotometer (UV-2600, Shimadzu, Kyoto, Japan) to study the solution binding of the gossypol. The morphologies of the samples were characterized by field emission scanning electron microscopy (FESEM) (SUPRA 55VP, Zeiss, Oberkochen, Germany). Nitrogen adsorption-desorption measurements were performed on an automated gas sorption analyzer (Autosorb-IQ, Quantachrome Instrument Corp, Boynton Beach, FL, America), and the specific surface areas (S) were evaluated by the Brunauer−Emmett-Teller (BET) method.

### 2.4. Binding Experiments

Binding studies were conducted to evaluate the adsorption performance of MIPs and NIPs for gossypol. In the study, the UV absorption peak at 373 nm was chosen for the quantitative analysis. By comparing the change of absorbance value before and after the uptake, the binding capacity of each material was obtained.

In the kinetic adsorption experiments, 10 mg of MIPs or NIPs were added to 5.0 mL of the gossypol solution in methanol at an initial concentration (*C*_0_, mg·L^−1^) of 300 mg·L^−1^ and were shaken for different time intervals ranging from 0 min to 900 min (MIP1 and NIP1) and 0 min to 90 min for MIP2, MIP3, NIP2, and NIP3. Then, the supernatants and polymers were separated using syringe filters (0.2 μm, PTFE), and the concentrations of gossypol in the supernatant (*C*_t_, mg·L^−1^) were measured by UV-Vis spectrometry following a reported procedure [9]. The adsorption capacity (*Q*_t_, mg·g^−1^) at given times was calculated according to Equation (1):

(1) $$Q_{t}=\frac{\left(C_{0}-C_{t}\right)V}{m}$$

where *V* (L) is the volume of gossypol solution, and *m* (g) is the weight of the absorbents.

In the adsorption isothermal experiments, 10 mg of MIPs or NIPs were added to 5.0 mL of gossypol solutions in methanol at various concentrations ranging from 200 mg·L^−1^ to 2000 mg·L^−1^ and were shaken for 12 h (MIP1 and NIP1). Ten mg of MIPs or NIPs were added to 5.0 mL of gossypol solutions in methanol at various concentrations ranging from 110 mg·L^−1^ to 1600 mg·L^−1^ for MIP2, MIP3, NIP2, and NIP3 and were shaken for 6 h to ensure adsorption equilibrium could be reached. The supernatants and polymers were separated by a syringe filter (0.2 μm, PTFE), and the supernatants were analyzed to determine the remaining concentration (*C*_e_, mg·L^−1^) using UV-Vis spectrometry (Shimadzu, Kyoto, Japan) [9]. The equilibrium adsorption capacity (*Q*_e_, mg·g^−1^) was calculated according to Equation (2):

(2) $$Q_{e}=\frac{\left(C_{0}-C_{e}\right)V}{m}$$

Two structurally similar analogs of ellagic acid and quercetin were chosen for the selectivity study. Ten mg of MIPs or NIPs were added to 5.0 mL of each analyte solution in methanol (200 mg·L^−1^ of gossypol, ellagic acid, and quercetin). After being shaken for 1 h, the supernatants and polymers were separated, and the residual concentrations of gossypol, ellagic acid, and quercetin were measured by UV-Vis spectrometry at 374, 366, and 371 nm, respectively. Additionally, the imprinting factor (IF) and selectivity coefficients (α) were used to evaluate the recognition selectivity of MIPs and NIPs towards gossypol and competitive compounds. IF and *α* were calculated using Equations (3) and (4):

(3) $$\mathrm{IF}=\frac{Q_{\mathrm{MIP}}}{Q_{\mathrm{NIP}}}$$

(4) $$\alpha =\frac{{\mathrm{IF}}_{T}}{{\mathrm{IF}}_{C}}$$

where *Q*_MIP_ (mg·g^−1^) and *Q*_NIP_ (mg·g^−1^) represent the adsorption capacity of the templates or analogs on MIPs and NIPs at the same conditions, respectively. IF_T_ and IF_C_ are the imprinting factors for gossypol and contrastive compounds, including ellagic acid and quercetin, respectively.

## 3. Results and Discussion

### 3.1. Preparation of the Gossypol-MIPs 

**Figure 1:**
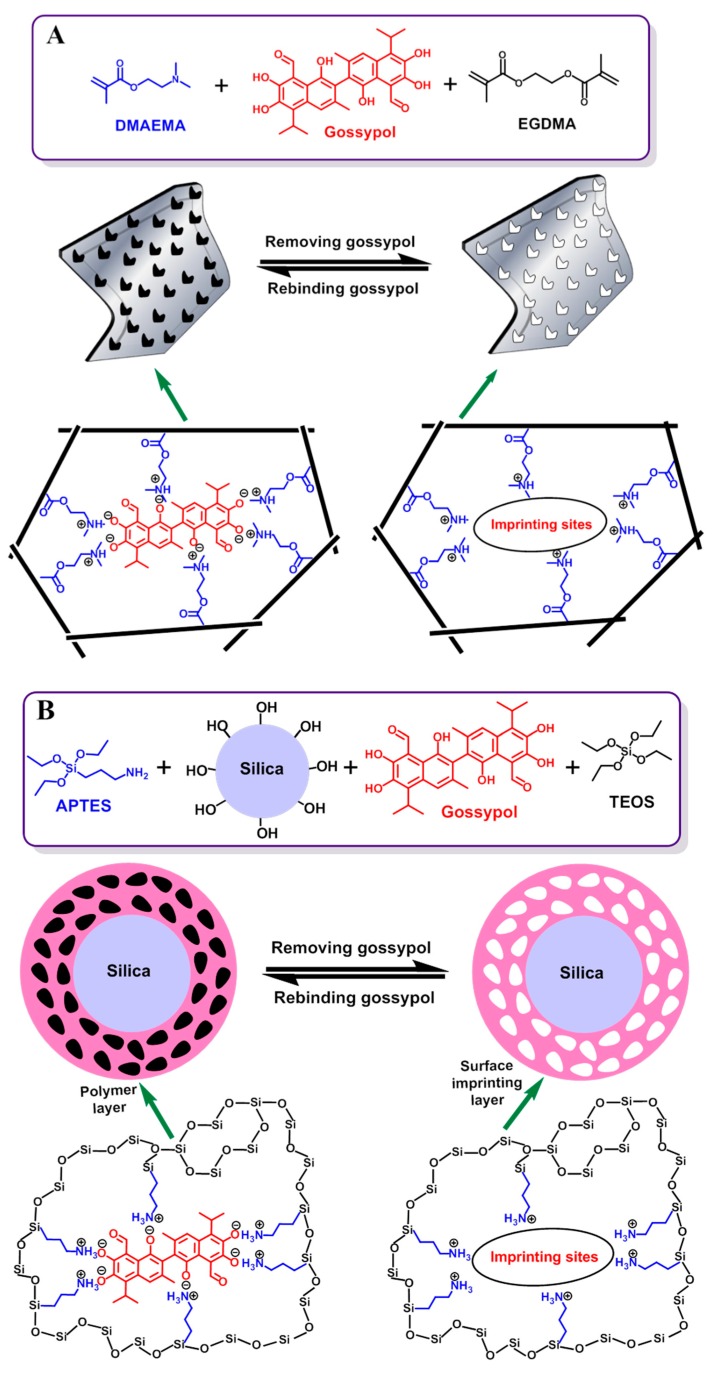
Schematic illustration of the synthetic procedure for gossypol-MIPs via bulk polymerization (**A**) and sol-gel surface imprinting (**B**).

The gossypol molecule is an acidic organic compound with p*K*a of 6.5 [50] containing six phenolic hydroxyl –OH functional groups in the structure. It can form an acid−base ionic pair interaction with the basic amino –NH_2_ group, which is beneficial for selectively binding and rebinding with the template. Theoretically, functional monomers containing basic groups could interact strongly with acidic phenolic hydroxyl groups via acid-base interaction. In the work reported here, two different routes were employed to prepare MIPs by bulk polymerization and the sol–gel process, combining a surface molecular imprinting protocol. The schematic procedure of the preparation of the gossypol MIPs is illustrated in Figure 1.

In our previous work [9], the bulk polymer was prepared using EGDMA as a cross-linker, DMAEMA as a functional monomer, and gossypol as the template. Compared with the bulk polymerization route in Figure 1A, the sol-gel surface layer imprinting is depicted in Figure 1B. Silica gel was chosen as the solid supporter for surface imprinting because it is non-toxic and stable under acidic conditions and elevated temperatures. Silica gel is an amorphous inorganic polymer that has siloxane groups (Si–O–Si) in the bulk and silanol groups (Si–OH) on its surface. The surface silanol groups facilitate the introduction of the organic groups, which covalently bind to the silane monomer. In order to increase the concentration of surface silanol groups, the activation of silica gel surface is necessary [47]. 

In the first step, silica gel particles were treated with methanesulfonic acid to increase the number of surface silanol groups [35]. In the next step, the complex was formed between the template gossypol and the functional monomer, APTES. It is noteworthy that APTES was the functional monomer capable of covalently bonding onto silica particles [36]. Then, the template-monomer complex was grafted onto the surface of the silica in the sol-gel process through the hydrolysis of silane and condensation with the activated silica gel in the presence of HAc and TEOS. TEOS acted as a cross-linking reagent, which was easily hydrolyzed in the presence of the HAc catalyst. This allowed the formation of a polymeric network on the surface of the SiO_2_. Finally, after removal of the template molecules from the surface imprinted layer, the MIP2 was obtained, leaving a large number of tailor-made cavities for gossypol at or close to the surface of the silica.

### 3.2. Characterization of MIPs and NIPs

#### 3.2.1. SEM Analysis

**Figure 2:**
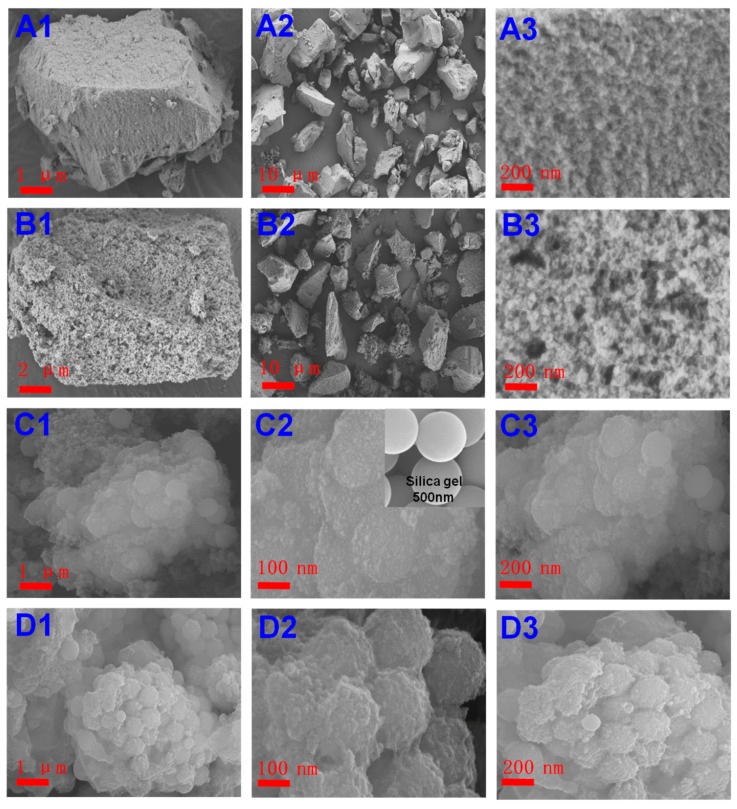
SEM images of non-imprinted polymer (NIP) and MIP (**A**) NIP1, (**B**) MIP1(**C**) NIP2; (**D**) MIP2.

The morphological structures of MIPs and NIPs prepared with different polymerization processes are shown in Figure 2. There were distinct differences between bulk polymerization and sol-gel surface layer imprinting. Both NIP1 (Figure 2A) and MIP1 (Figure 2B) were prepared by bulk polymerization. After the prepared MIPs and NIPs were grinded, heterogeneous particles were obtained with irregular shapes and different sizes. Compared to the surface morphology of NIP1 (Figure 2A1–A3), MIP1 (Figure 2B1–B3) was rough, loose, and porous. The imprinted recognition sites within cavities could serve as predetermined high affinity sites for the template. From Figure 2C,D, it was observed that by choosing the appropriate silica-gel support in terms of size and shape, the MIPs via surface layer imprinting were roughly spherical in shape. It was obvious that the morphologies of the MIP2 (Figure 2D) and the NIP2 (Figure 2C) were completely different from the morphology of the activated silica particles. Result showed that the surface of the silica gel was quite smooth, while the MIPs exhibited a highly rough outside polymeric surface, larger particle size, and a certain degree of agglomeration, indicating the formation of the imprinted layer on the surface of the silica support and the successful synthesis of the surface imprinted polymers. After the imprinting process (the support silica gel with the average particle size of approximately 500 nm), the average diameter of the MIP2 increased to approximately 900 nm, as shown in Figure 2D3, implying that a 200 nm thickness imprinting layer was formed on the surface of the silica gel. Similar surface morphologies were observed for MIPs and NIPs.

#### 3.2.2. Surface Area Measurement via Nitrogen Adsorption-Desorption Analysis 

The surface area of MIPs and NIPs were measured with nitrogen adsorption-desorption analysis. Listed in Table 2 are the structure parameters of MIPs, NIPs, and their silica substrate, including the surface area and average pore diameter. Results showed that the specific surface area for silica gel was only 14.29 m^2^ g^−1^. In comparison with silica gel, there was a significant increase of surface area for MIP2 (268.2 m^2^ g^−1^) and NIP2 (31.53 m^2^ g^−1^). However, for bulk polymerization, MIP1 had much lower (seven times lower) surface area compared to that of NIP1 (13.9 m^2^ g^−1^ versus 91.1 m^2^ g^−1^). This implied surface layer imprinting was beneficial for increasing the specific surface area for molecular imprinting. By sol-gel surface layer imprinting, the specific surface area of MIP2 was seven times higher than that of NIP2. 

### 3.3. Adsorption Kinetics of MIPs and NIPs 

**Figure 3:**
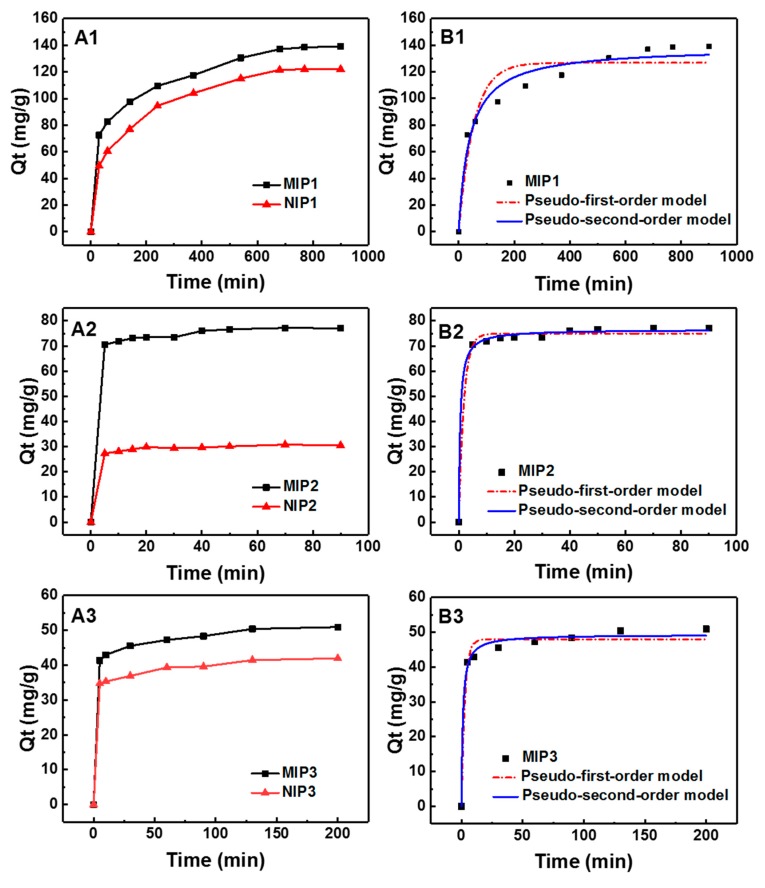
(**A**) Adsorption kinetics of MIPs and NIPs; (**B**) two kinetic models by nonlinear fitting for MIPs (**A1**: bulk polymerization: 10 mg of absorbents in 5.0 mL of 300 mg·L^−1^ gossypol solution from 0 to 900 min; **A2**: sol−gel and surface layer imprinting: 10 mg of absorbents in 5.0 mL of 300 mg·L^−1^ gossypol solution from 0 to 90 min; **A3**: sol−gel without adding silica gel: 10 mg of absorbents in 5.0 mL of 300 mg·L^−1^ gossypol in methanol from 0 to 200 min).

Adsorption kinetic experiments for MIP1, MIP2, MIP3, NIP1, NIP2, and NIP3 were conducted to study the recognition behavior of MIPs and NIPs. For MIP1 and NIP1, the adsorption kinetic studies were carried out by using 300 mg·L^−1^ gossypol in methanol at different adsorption time intervals ranging from 0 min to 900 min. Figure 3A1 shows that the adsorption capacity of MIP1 increased rapidly in the first 60 min, then slowed down and eventually reached adsorption equilibrium in 720 min. This result implied slow binding kinetics of the recognition process for MIPs prepared via bulk polymerization. The adsorption kinetic experiments were carried for MIP2 and NIP2, which were prepared by sol−gel surface layer imprinting using a 300 mg·L^−1^ gossypol solution in methanol at different adsorption times ranging from 0 min to 90 min. Figure 3A2 shows the adsorption capacity increased rapidly in the first 5 min and quickly reached equilibrium at 40 min, demonstrating a fairly rapid adsorption kinetics. At the early stage of the adsorption, MIP2 possessed a large amount of empty recognition sites within the surface imprinted layers, which enabled gossypol to be easily adsorbed onto those sites with less resistance. Over time, the adsorption rate slowed down because the bound gossypol had occupied most of the binding sites; eventually, the adsorption reached equilibrium. Such rapid adsorption was desirable for its application. For the sol−gel process without adding the silica gel support, adsorption kinetic experiments were carried out by using a 300 mg L^−1^ gossypol solution in methanol for MIP3 and NIP3 at time intervals of 0 to 200 min. Figure 3A3 shows the overall kinetic curve of MIP3 was similar to that of MIP2 but with much lower adsorption capacity than that of MIP3.

The equilibrium times, adsorption capacities, and IF of MIP1, MIP2, and MIP3 are summarized in Table 3. For MIP1, it took as long as 720 min to reach equilibrium. However, for MIP2 and MIP3, the adsorption equilibrium time was much shorter. Results showed that the sol-gel and surface layer imprinting promoted the diffusion of the template to the binding sites. The consequence of this was more complete removal of templates and lower mass transfer resistance compared to traditional bulk MIPs. The differences in kinetic and *Q*_e_ between MIP1 and MIP2 could also be related to the different affinity of monomers used for MIP synthesis—not only to morphological aspects.

In addition, the IF value of MIP2 was 2.53, which was much higher than that of MIP1 (IF = 1.14) and MIP3 (IF = 1.22), indicating a better imprinting effect of the surface molecular imprinting. Overall, the faster adsorption kinetics, decent binding capacity, and improved imprinting effect demonstrated the merits of silica gel as the supporting material in the preparation of molecular imprinted materials.

A comparison of the kinetics of adsorption behaviors of MIPs and NIPs by different imprinting approaches for gossypol is presented in Figure 3B1–B3), and the corresponding kinetic parameters and correlation coefficients are summarized in Table 4. The two most common kinetic models—the pseudo-first-order and the pseudo-second-order models [51,52]—were employed to analyze the kinetic data. The pseudo-first-order and pseudo-second-order rate equations were given as follows:

(5) $$\ln\left(Q_{e}-Q_{t}\right)=\ln Q_{e}-k_{1}t$$

(6) $$\frac{t}{Q_{t}}=\frac{1}{k_{2}Q_{e}^{2}}+\frac{t}{Q_{e}}$$

where *Q*_e_ (mg g^−1^) and *Q*_t_ (mg g^−1^) are the binding capacity of gossypol adsorbed at equilibrium and at a specific time t (min), respectively; k_1_ (min^−1^) is the pseudo-first-order rate constant of adsorption, and k_2_ (g mg^−1^ min^−1^) is the rate constant of the pseudo-second-order adsorption model.

According to the data in Table 4, the regression values *R*^2^ of the pseudo-second-order model were higher than those of the pseudo-first-order models, indicating which fit better for describing the gossypol binding process of MIPs. Furthermore, the theoretical adsorption capacity (*Q*_e_, cal) value calculated from the pseudo-second-order kinetic model was in good agreement with the experimental adsorption capacity (*Q*_e_, exp) data. Thus, the pseudo-second-order mechanism was predominant, and the chemisorption was probably the rate-limiting step controlling the gossypol-binding process [53].

### 3.4. Adsorption Isotherms of MIPs

**Figure 4:**
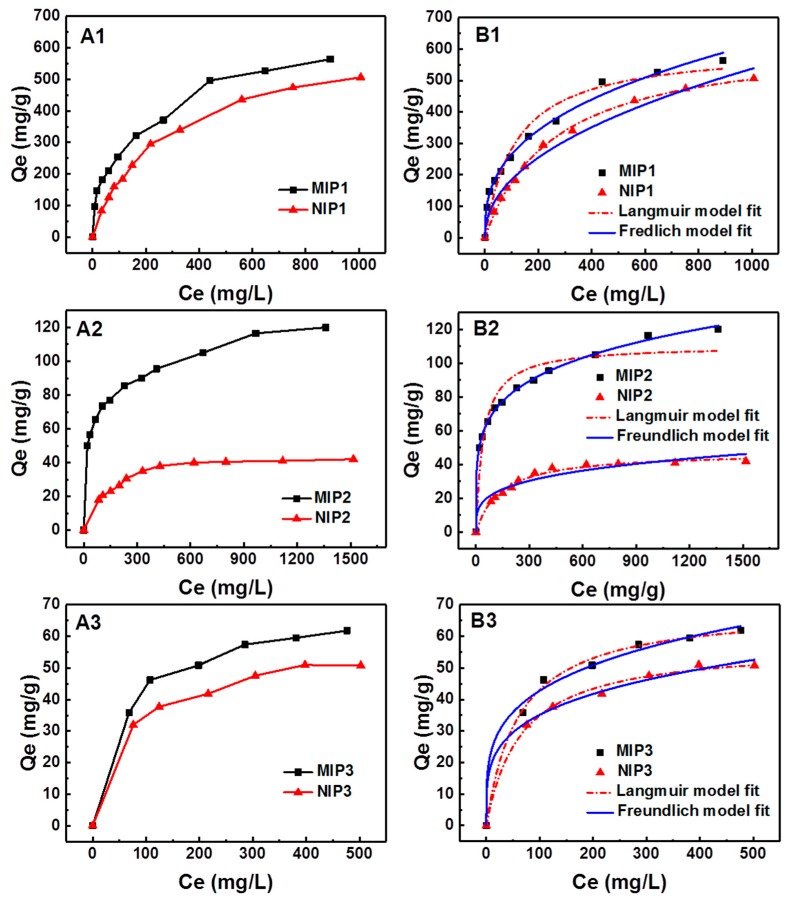
(**A**) Adsorption isotherms of MIPs and NIPs for gossypol; (**B**) evaluating binding models by nonlinear fitting of binding isotherm for MIPs and NIPs for gossypol (**A1**, **A2,** and **A3**: 10 mg of absorbents in 5.0 mL gossypol in methanol at different concentration for 12 h).

The adsorption isotherms of MIPs and NIPs are presented in Figure 4A. The adsorption isotherm was obtained via the binding experiment using 10 mg of the MIP1 and NIP1 with the gossypol methanol solution. As shown in Figure 4A1, the gossypol adsorption capacity of MIPs and NIPs both increased at equilibrium time with increasing concentration of gossypol. The binding capacity of MIPs was 13–15% higher than that of NIPs for gossypol. Results supported the hypothesis that imprinted sites were indeed generated and were beneficial for gossypol recognition and rebinding in MIPs.

The binding isotherm was also obtained to establish the relationship between the binding capacity and equilibrium concentration for MIP2. The equilibrium adsorption capacity increased with increasing initial concentration of gossypol (Figure 4A2), which was reasonable considering the concentration gradient could be an effective driving force that would propel the diffusion of the template gossypol into the channels and cavities in MIP2 [32]. The amount of gossypol adsorbed by MIP2 once it reached saturation was 120 mg g^−1^ at 1600 mg L^−1^ gossypol concentration. The absorption isotherm of NIP2 was also obtained. The saturated adsorption capacity was 42 mg g^−1^, which was much lower than that of MIP2. This result supported the formation of the gossypol imprinted sites in MIP2 compared to the control of NIP2. Additionally, MIP2 showed significantly higher adsorption capacity compared to that of NIP2 throughout the whole concentration range. This indicated that the molecularly imprinted polymer exhibited a strong memory effect and excellent imprinting for template gossypol. 

The adsorption capacities of MIP3 and NIP3 were compared with MIP1, NIP1, MIP2, and NIP2. As shown in Figure 4A3, when the MIP3 was made via the sol-gel process without adding the silica gel support, the difference in adsorption capacity between MIP3 and NIP3 was relatively small. MIP3 still exhibited a relatively higher adsorption capacity than that of NIP3, suggesting the formation of imprinted sites. The maximum adsorption capacities of MIP3 and NIP3 for gossypol were 61.8 mg g^−1^ and 51.8 mg g^−1^, which were much lower than those of MIP1 (*Q*_exp_ = 564 mg g^−1^) and MIP2 (*Q*_exp_ = 120 mg g^−1^). 

The binding data were fitted with the Langmuir model and the Freundlich model [54,55]. The Langmuir model assumed that the adsorption took place on a homogeneous surface with monolayer coverage. The binding sites were also assumed to be energetically equivalent, and there were no interactions between molecules adsorbed on adjacent sites. The equation of the Langmuir model is described as follows:

(7) $$Q_{e}=\frac{Q_{m}K_{L}C_{e}}{1+K_{L}C_{e}}$$

where *Q*_e_ (mg g^−1^) is the amount of gossypol adsorbed on the adsorbent at equilibrium, *C*_e_ (mg L^−1^) is the free gossypol concentration in the solution at equilibrium (mg L^−1^), *Q*_m_ (mg g^−1^) is the maximum adsorption capacity of the adsorbent, And *K*_L_ (L mg^−1^) is the Langmuir constant, which is related to the affinity of the binding sites.

The Freundlich model was an exponential equation that described reversible adsorption and was suitable for multilayer adsorption of a heterogeneous system and not restricted to the formation of the monolayer. It took the form as Equation (8):

(8) $$Q_{e}=K_{F}C_{e}^{1/n}$$

where *K*_F_ (mg g^−1^) and n are both the Freundlich constants, which represent the adsorption capacity and adsorption favorability of the system, respectively. If *n* > 1, it suggests favorable adsorption and increased adsorption capacity.

The binding data of adsorption for gossypol were fitted with the Langmuir and the Freundlich models by nonlinear regression. The nonlinear regression plots of the two models are shown in Figure 4B, and the parameters calculated from isotherm models are listed in Table 5. The applicability of the isotherm models to the adsorption behaviors was evaluated based on the correlation coefficient (R^2^). Results showed that the Freundlich model better fit the experimental data of gossypol on MIP2 than the Langmuir model. It implied that the binding site configurations in MIP1 and MIP2 were heterogeneous in respect to the affinity for gossypol. However, the Langmuir model was found to better fit the experimental binding data of gossypol on MIP3. Results demonstrated that high-affinity binding sites and low-affinity binding sites coexisted in MIP1 and MIP2, and for MIP3, binding occurred on a homogeneous surface by monomolecular layer sorption. Additionally, the Langmuir model yielded a better fit for NIP1 and NIP2, indicating the adsorptions of gossypol onto the NIP2 could be monolayer adsorption. Noticeably, the adsorption of NIP3 could be better described by the Freundlich model.

The Scatchard equation was adopted to evaluate the binding ability of different sites in MIPs and NIPs. The Scatchard equation is expressed as follows:

(9) $$\frac{Q_{e}}{C_{e}}=\frac{Q_{m}-Q_{e}}{k_{D}}$$

*Q*_e_ (mg g^−1^) and *C*_e_ (mg L^−1^) are the equilibrium adsorption capacity and the equilibrium concentration of gossypol, *Q*_m_ (mg g^−1^) is the maximum adsorption capacity of the MIP, and KD (mg L^−1^) is the equilibrium dissociation constant. 

**Figure 5:**
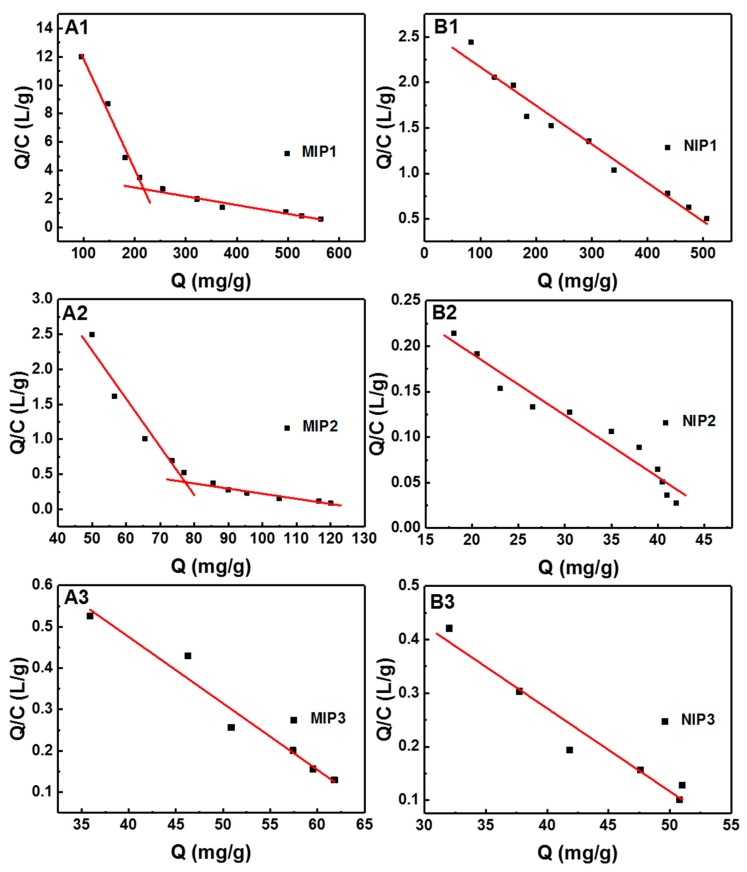
(**A**) Scatchard plot of MIPs for gossypol; (**B**) Scatchard plot of MIPs for gossypol.

The binding isotherm was used to differentiate recognition sites in terms of their binding ability using the Scatchard model (Figure 5). The Scatchard plot for MIP1 and MIP2 were two lines with different slopes, indicating binding site heterogeneity in MIPs. However, the Scatchard plot for MIP3 was a single straight line, implying that the binding sites in the MIP3 were almost homogeneous. The Scatchard plots for MIP1 and MIP2 contained two different linear regression lines, suggesting two types of binding sites, e.g., higher-affinity and lower-affinity binding sites. Linear regression parameters of the Scatchard plot are summarized in Table 6. As can be seen, the higher-affinity binding sites dominated at a lower concentration range of 200–500 mg L^−1^, while the lower-affinity binding sites prevailed at a higher concentration range of 500–2000 mg L^−1^. The Q_max_ values at higher concentrations were 654 mg g^−1^. The experimental Q_e_ values at the highest concentration were 564 mg g^−1^. Such high binding capacity also demonstrated that the prepared MIP1 had outstanding binding affinity towards the target template gossypol.

### 3.5. Adsorption Selectivity of the MIPs

**Figure 6:**
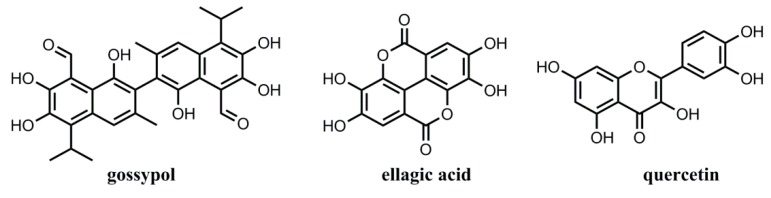
Structures of template gossypol, ellagic acid, and quercetin.

**Figure 7:**
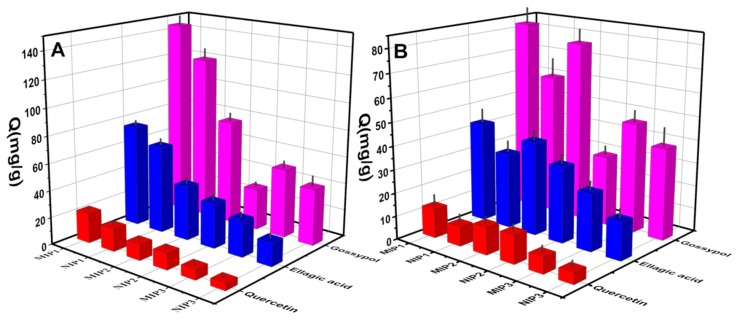
The selective adsorption capacities of the MIP1, MIP2, MIP3, NIP1, NIP2, and NIP3 toward gossypol, ellagic acid, and quercetin solution, respectively (**A**: 10 mg of absorbents in 5.0 mL 200 mg L^−1^ template solution in methanol for 12 h; **B**: 10 mg of absorbents in 5.0 mL 200 mg L^−1^ template solution in methanol for 1 h).

To evaluate the binding specificity of the prepared MIPs and NIPs toward gossypol, an adsorption selectivity experiment was conducted using structurally similar analogues of ellagic acid and quercetin as control templates (Figure 6). Figure 7 presents the selective adsorption capacities of MIPs and NIPs for gossypol and two competitors at concentrations of 200 mg L^−1^ for 1 h and 12 h, respectively. Results showed that the adsorption capacity of MIP1 for gossypol was about 1.9 times higher than that of MIP2 and 2.8 times higher than that of MIP3 for the 12 h binding experiment. However, these values of MIP1 and MIP2 were found to be close for the 1 h binding experiment. The result was consistent with the fact that MIP2 had fast binding kinetics due to the advantage of surface layer imprinting, while the MIP1 featured high binding capacity with long equilibrium time. Moreover, the adsorption capacities of MIP1, MIP2, and MIP3 for gossypol were much higher than those of the other two analogues, suggesting that MIP1, MIP2, and MIP3 have higher affinities for the template gossypol. The adsorption capacity data was summarized in Table 7.

## 4. Conclusions

In summary, three sets of MIPs for gossypol were prepared by bulk polymerization (MIP1), sol-gel surface layer imprinting (MIP2), and a sol−gel process (MIP3). MIP1 showed the highest adsorption capacity up to 564 mg g^−1^. The obtained MIP2 and MIP3 exhibited faster adsorption kinetics towards gossypol compared with MIP1. Noticeably, MIP2 prepared with sol-gel surface layer imprinting combined the merits of the sol−gel process and surface imprinting using the silica gel as the supporting material. It only took 40 min for MIP2 to reach adsorption equilibrium. The selectivity of MIPs was also satisfactory. All above mentioned results suggested the MIP2 was suitable as a sorbent for rapid recognition and separation for gossypol, and MIP1 was desirable for gossypol binding at high concentrations. Both types of MIPs had potential value in different practical applications, such as the removal of gossypol from cotton seed, in food safety control, and drug monitoring.

## Figures and Tables

**Table 1:** Polymerization conditions for the preparation of bulk polymerization and sol-gel surface layer imprinting.

Polymer	Template (mmol)	FM (mmol)	Cross-linker (mmol)	Initiator/Catalyst	Support (g)	Solvent (mL)
MIP1 ^a^	Gossypol (0.083)	DMAEMA (1.0)	EGDMA (5.0)	AIBN (0.27 mmol)		CH_2_Cl_2_ (4.0)
NIP1 ^a^		DMAEMA (1.0)	EGDMA (5.0)	AIBN (0.27 mmol)		CH_2_Cl_2_ (4.0)
MIP2 ^b^	Gossypol (0.083)	APTES (1.25)	TEOS (2.6)	1.0 mol L^−1^ HAc (0.15mL)	activated silica (0.058)	Acetone (3.0)
NIP2 ^b^		APTES (1.25)	TEOS (2.6)	1.0 mol L^−1^ HAc (0.15mL)	activated silica (0.058)	Acetone (3.0)
MIP3 ^b^	Gossypol (0.083)	APTES (1.25)	TEOS (2.6)	1.0 mol L^−1^ HAc (0.15mL)		Acetone (3.0)
NIP3 ^b^		APTES (1.25)	TEOS (2.6)	1.0 mol L^−1^ HAc (0.15mL)		Acetone (3.0)

^a^: bulk polymerization carried out at 65 °C for 24 h; ^b^: sol−gel surface imprinting and sol-gel process carried out at room temperature (RT) for 24 h. * NIP = non-imprinted polyer, MIP = molecularly imprinted polymers, DMAEMA = dimethylaminoethyl methacrylate, EGDMA = ethylene glycol dimethacrylate, AIBN = 2,2-azobis(isobutyronitrile), APTES = (3-aminopropyl) triethoxysilane, TEOS = tetraethoxysilane.

**Table 2:** **Brunauer−Emmett-Teller** BET surface area and average pore diameter of MIPs and NIPs and silica gel supports.

Samples	BET Surface Area (m^2^ g^−1^)	Average Pore Diameter (nm)
NIP1	91.1	6.57
MIP1	13.9	40.4
Silica gel (spherical 500 nm)	14.29	23.07
NIP2	31.53	22.29
MIP2	268.2	5.542

**Table 3:** Adsorption equilibrium times (*t*_e_, min), adsorption capacities at equilibrium (*Q*_e_, mg·g^−1^), and imprinting factors (IF) of MIP 1, MIP2, and MIP3.

Samples	*t*_e_ (min)	*Q*_MIP-e_ (mg·g^−1^)	*Q*_NIP-e_ (mg·g^−1^)	IF
MIP1	720	139.00	122.00	1.14
MIP2	40	77.05	30.50	2.53
MIP3	140	50.95	41.95	1.22

**Table 4:** Kinetic parameters for the adsorption of gossypol onto MIPs and NIPs prepared on 60 μm spherical silica gel support.

Sample	*Q*_e_, exp (mg g^−1^)	Pseudo-First-Order			Pseudo -Second-Order		
		*Q*_e_, cal (mg g^−1^)	*k*_1_ (min^−1^)	*R* ^2^	*Q*_e_ (mg g^−1^)	*k*_2_ (g mg^−1^ min^−1^)	*R* ^2^
MIP1	139.00	126.82	0.01869	0.9006	138.56	1.84×10^−4^	0.9633
MIP2	77.05	74.94	0.5490	0.9936	76.54	0.02523	0.9976
MIP3	50.95	39.36	0.3924	0.9750	49.30	0.01772	0.9915

**Table 5:** Model fitting parameters from binding isotherm of MIPs and NIPs for gossypol.

Sample	*Q*_e,exp_ (mg g^−1^)	Langmuir			Freundlich		
		*Q*_m_ (mg g^−1^)	*K*_L_ (L mg^−1^)	*R* ^2^	*K*_F_ (mg g^−1^)	*n*	*R* ^2^
MIP1	564	606.9	0.00869	0.9527	27.54	2.751	0.9926
NIP1	506.5	635.3	0.00384	0.9979	49.78	2.155	0.9838
MIP2	120	110.6	0.02401	0.9287	27.54	4.842	0.9984
NIP2	42	47.37	0.00725	0.9891	6.915	3.853	0.9357
MIP3	61.8	69.02	0.01661	0.9959	13.53	4.000	0.9923
NIP3	50.8	57.50	0.01543	0.9943	11.15	4.013	0.9958

**Table 6:** Adsorption parameters from the Scatchard analysis of MIPs and NIPs.

Samples	*C*_0_ (mg L^−1^)	Regression Equation Y = A + BX	*K*_D_ (mg L^−1^) *K*_D_ = −1/B	*Q*_m_ (mg g^−1^) *Q*_m_ = −A/B	*R* ^2^
MIP1	200–500	*Q*_e_/*C*_e_ = 19.6 − 0.0776Q	12.9	252	0.9761
	600–2000	*Q*_e_/*C*_e_ = 4.04 − 0.00617Q	162.1	654	0.9222
NIP1	200–2000	*Q*_e_/*C*_e_ = 2.59 − 0.0042Q	238.1	616.7	0.9675
MIP2	120–260	*Q*_e_/*C*_e_ = 5.71 − 0.0688Q	14.5	83.0	0.9223
	300–1600	*Q*_e_/*C*_e_ = 0.95 − 0.00730Q	137.0	130.1	0.9099
NIP2	120–1600	*Q*_e_/*C*_e_ = 0.33 − 0.0068Q	147.1	48.5	0.9493
MIP3	140–600	*Q*_e_/*C*_e_ = 1.12 − 0.0161Q	62.1	69.6	0.9477
NIP3	140–600	*Q*_e_/*C*_e_ = 0.89 − 0.0155Q	64.5	57.4	0.9355

**Table 7:** The adsorption capacities of gossypol, ellagic acid, and quercetin of MIP and NIP.

*Q* (mg g^−1^)	Adsorbates (12 h)			Adsorbates (1 h)		
	Gossypol	Ellagic Acid	Quercetin	Gossypol	Ellagic Acid	Quercetin
MIP1	144.5	75	21.9	82.5	42.8	12.5
NIP1	121.1	64.5	16.7	60.5	32.2	8.3
MIP2	77.3	40.1	11.7	77.0	40.1	11.7
NIP2	30.5	33.2	12.1	30.1	32.7	12.1
MIP3	50.9	26.5	7.7	47.2	24.6	7.1
NIP3	41.8	17.3	5.3	39.3	16.3	4.9

## References

[1] Withers W.A., Carruth F.E. (1915). Gossypol—A toxic substance in cottonseed. A preliminary note. Science.

[2] Adams R., Geissman T.A., Edwards J.D. (1960). Gossypol, a pigment of cottonseed. Chem. Rev..

[3] Maugh T.H. (1981). Male “pill” blocks sperm enzyme. Science.

[4] Randel R.D., Chase C.C., Wyse S.J. (1992). Effects of gossypol and cottonseed products on reproduction of mammals. J. Anim. Sci..

[5] Montamat E.E., Burgos C., De Burgos N.M.G., Rovai L.E., Blanco A., Segura E.L. (1982). Inhibitory action of gossypol on enzymes and growth of Trypanosoma cruzi. Science.

[6] Zhang W., Xu Z., Zhao S., Sun J., Yang X. (2007). Development of a microbial fermentation process for detoxification of gossypol in cottonseed meal. Anim. Feed. Sci. Technol..

[7] Johnson L.A., Lusas E.W. (1983). Comparison of alternative solvents for oils extraction. J. Am. Oil. Chem. Soc..

[8] Barraza M.L., Coppock C.E., Brooks K.N., Wilks D.L., Saunders R.G., Latimer G.W. (1991). Iron sulfate and feed pelleting to detoxify free gossypol in cottonseed diets for dairy cattle. J. Dairy Sci..

[9] Zhi K., Wang L., Zhang Y., Zhang X., Zhang L., Liu L., Yao J., Xiang W. (2018). Preparation and evaluation of molecularly imprinted polymer for selective recognition and adsorption of gossypol. J. Mol. Recognit..

[10] Bai L., Romanova E.V., Sweedler J.V. (2011). Distinguishing Endogenous d-Amino Acid-Containing Neuropeptides in Individual Neurons Using Tandem Mass Spectrometry. Anal. Chem..

[11] Kuk M.S., Tetlow R. (2005). Gossypol removal by adsorption from cottonseed miscella. J. Am. Oil. Chem. Soc..

[12] Kuk M.S., Hronsr R.J., Abraham G. (1993). Adsorptive gossypol removal. J. Am. Oil. Chem. Soc..

[13] Wuff G., Sarhan A. (1972). The use of polymers with enzyme-analogous structures for the resolution of racemate. Angew. Chem. Int. Ed..

[14] Andersson L., Sellergren B., Mosbach K. (1984). Imprinting of amino acid derivatives in macroporous polymers. Tetrahedron Lett..

[15] Sellergren B., Shea K.J. (1993). Influence of polymer morphology on the ability of imprinted network polymers to resolve enantiomers. J. Chromatogr. A.

[16] Vlatakis G., Andersson L.I., Müller R., Mosbach K. (1993). Drug assay using antibody mimics made by molecular imprinting. Nature.

[17] Whitcombe M.J., Rodriguez M.E., Villar P., Vulfson E.N. (1995). A new method for the introduction of recognition site functionality into polymers prepared by molecular imprinting: Synthesis and characterization of polymeric receptors for cholesterol. J. Am. Chem. Soc..

[18] Mosbach K. (1992). Preparation of Synthetic Enzymes and Synthetic Antibodies and Use of the Thus Prepared Enzymes and Antibodies. US Patent.

[19] Zhu J.L., Chen D.P., Ai Y.H., Dang X.P., Huang J.L., Chen H.X. (2017). A dummy molecularly imprinted monolith for selective solid-phase microextraction of vanillin and methyl vanillin prior to their determination by HPLC. Microchim. Acta.

[20] Deiminiat B., Rounaghi G.H., Arbab-Zavar M.H. (2017). Development of a new electrochemical imprinted sensor based on poly-pyrrole, sol-gel and multiwall carbon nanotubes for determination of tramadol. Sens. Actuators B.

[21] Li C.Y., Ma X.G., Zhang X.J., Wang R., Chen Y., Li Z.Y. (2016). Magnetic molecularly imprinted polymer nanoparticles-based solid-phase extraction coupled with gas chromatography-mass spectrometry for selective determination of trace di-(2-ethylhexyl) phthalate in water samples. Anal. Bioanal. Chem..

[22] Chen L., Wang X., Lu W., Wu X., Li J. (2016). Molecular imprinting: Perspectives and applications. Chem. Soc. Rev..

[23] Wulff G., Liu J. (2012). Design of biomimetic catalysts by molecular imprinting in synthetic polymers: The role of transition state stabilization. Acc. Chem. Res..

[24] Whitcombe M.J., Chianella I., Larcombe L., Piletsky S.A., Noble J., Porter R., Horgan A. (2011). The rational development of molecularly imprinted polymer-based sensors for protein detection. Chem. Soc. Rev..

[25] Chen L., Xu S., Li J. (2011). Recent advances in molecular imprinting technology: Current status, challenges and highlighted applications. Chem. Soc. Rev..

[26] Yin J., Cui Y., Yang G., Wang H. (2010). Molecularly imprinted nanotubes for enantioselective drug delivery and controlled release. Chem. Commun..

[27] Qin L., Jia X., Yang Y., Liu X. (2016). Porous Carbon Microspheres: An Excellent Support To Prepare Surface Molecularly Imprinted Polymers for Selective Removal of Dibenzothiophene in Fuel Oil. Ind. Eng. Chem. Res..

[28] Moczko E., Guerreiro A., Piletska E., Piletsky S. (2013). PEG-Stabilized Core–Shell Surface-Imprinted Nanoparticles. Langmuir.

[29] Jia X., Xu M., Wang Y., Ran D., Yang S., Zhang M. (2013). Polydopamine-based molecular imprinting on silica-modified magnetic nanoparticles for recognition and separation of bovine hemoglobin. Analyst.

[30] He Y., Huang Y., Jin Y., Liu X., Liu G., Zhao R. (2014). Well-Defined Nanostructured Surface-Imprinted Polymers for Highly Selective Magnetic Separation of Fluoroquinolones in Human Urine. ACS Appl. Mater. Interfaces.

[31] Gao R., Mu X., Zhang J., Tang Y. (2014). Specific recognition of bovine serum albumin using superparamagnetic molecularly imprinted nanomaterials prepared by two-stage core–shell sol–gel polymerization. J. Mater. Chem. B.

[32] Duan F., Chen C., Chen L., Sun Y., Wang Y., Yang Y., Liu X., Qin Y. (2014). Preparation and Evaluation of Water-Compatible Surface Molecularly Imprinted Polymers for Selective Adsorption of Bisphenol A from Aqueous Solution. Ind. Eng. Chem. Res..

[33] Qin Y.P., Li D.Y., He X.W., Li W.Y., Zhang Y.K. (2016). Preparation of High-Efficiency Cytochrome c-Imprinted Polymer on the Surface of Magnetic Carbon Nanotubes by Epitope Approach via Metal Chelation and Six-Membered Ring. ACS Appl. Mater. Interfaces.

[34] Arfaoui F., Khlifi A., Bargaoui M., Khalfaoui M., Kalfat R. (2018). Thin Melamine Imprinted Sol Gel Coating on Silica Beads: Experimental and Statistical Physics Study. Chem. Afr..

[35] Chrzanowska A.M., Poliwoda A., Wieczorek P.P. (2015). Surface molecularly imprinted silica for selective solid-phase extraction of biochanin A, daidzein and genistein from urine samples. J. Chromatogr. A.

[36] Meng M., Wang Z., Ma L., Zhang M., Wang J., Dai X., Yan Y. (2012). Selective Adsorption of Methylparaben by Submicrosized Molecularly Imprinted Polymer: Batch and Dynamic Flow Mode Studies. Ind. Eng. Chem. Res..

[37] Ren Y., Ma W., Ma J., Wen Q., Wang J., Zhao F. (2012). Synthesis and properties of bisphenol A molecular imprinted particle for selective recognition of BPA from water. J. Colloid Interface Sci..

[38] Wang S., Xu Z., Fang G., Duan Z., Zhang Y., Chen S. (2007). Synthesis and characterization of a molecularly imprinted silica gel sorbent for the on-line determination of trace Sudan I in Chilli powder through high-performance liquid chromatography. J. Agric. Food Chem..

[39] Zhu R., Zhao W., Zhai M., Wei F., Cai Z., Sheng N., Hu Q. (2010). Molecularly imprinted layer-coated silica nanoparticles for selective solid-phase extraction of bisphenol A from chemical cleansing and cosmetics samples. Anal. Chim. Acta.

[40] Zhao C., Wu D. (2013). Rapid detection assay for the molecular imprinting of gossypol using a two-layer PMAA/SiO2 bulk structure with a piezoelectric imprinting sensor. Sens. Actuators B.

[41] Zhi K., Wang L., Zhang Y., Jiang Y., Zhang L., Yasin A. (2018). Influence of Size and Shape of Silica Supports on the Sol–Gel Surface Molecularly Imprinted Polymers for Selective Adsorption of Gossypol. Materials.

[42] Arabi M., Ghaedi M., Ostovan A. (2017). Development of a Lower Toxic Approach Based on Green Synthesis of Water-Compatible Molecularly Imprinted Nanoparticles for the Extraction of Hydrochlorothiazide from Human Urine. ACS Sustain. Chem. Eng..

[43] Luo J., Gao Y., Tan K., Wei W., Liu X. (2016). Preparation of a Magnetic Molecularly Imprinted Graphene Composite Highly Adsorbent for 4-Nitrophenol in Aqueous Medium. ACS Sustain. Chem. Eng..

[44] Lofgreen J.E., Ozin G.A. (2014). Controlling morphology and porosity to improve performance of molecularly imprinted sol-gel silica. Chem. Soc. Rev..

[45] Zhang Z., Li J., Wang X., Shen D., Chen L. (2015). Quantum Dots Based Mesoporous Structured Imprinting Microspheres for the Sensitive Fluorescent Detection of Phycocyanin. ACS Appl. Mater. Interfaces.

[46] Wang Y., Yang Y., Xu L., Zhang J. (2011). Bisphenol A sensing based on surface molecularly imprinted, ordered mesoporous silica. Electrochim. Acta.

[47] Jiang X., Tian W., Zhao C., Zhang H., Liu M. (2007). A novel sol-gel-material prepared by a surface imprinting technique for the selective solid-phase extraction of bisphenol A. Talanta.

[48] Dai J., Zhang Y., Pan M., Kong L., Wang S. (2014). Development and Application of Quartz Crystal Microbalance Sensor Based on Novel Molecularly Imprinted Sol–Gel Polymer for Rapid Detection of Histamine in Foods. J. Agric. Food Chem..

[49] Xu S., Lu H., Li J., Song X., Wang A., Chen L., Han S. (2013). Dummy Molecularly Imprinted Polymers-Capped CdTe Quantum Dots for the Fluorescent Sensing of 2,4,6-Trinitrotoluene. ACS Appl. Mater. Interfaces.

[50] Reyes J., Wyrick S.D., Borriero L., Benos D.J. (1986). Membrane actions of male contraceptive gossypol tautomers. BBA Biomembr..

[51] Ho Y.S., McKay G. (1999). Pseudo-second order model for sorption processes. Process Biochem..

[52] Ho Y.S. (2006). Review of second-order models for adsorption systems. J. Hazard. Mater..

[53] Gao R., Mu X., Hao Y., Zhang L., Zhang J., Tang Y. (2014). Combination of surface imprinting and immobilized template techniques for preparation of core–shell molecularly imprinted polymers based on directly amino-modified Fe3O4 nanoparticles for specific recognition of bovine hemoglobin. J. Mater. Chem. B.

[54] Langmuir I. (1916). The constitution and fundamental properties of solids and liquids. Part I. Solids. J. Am. Chem. Soc..

[55] Freundlich H. (1906). Over the adsorption in solution. J. Phys. Chem. B.

